# Utilizing metakaolin and siliceous waste from the alum industry to create geopolymer adsorbent for the removal of certain heavy metals

**DOI:** 10.1038/s41598-025-10800-w

**Published:** 2025-07-28

**Authors:** Khaled Elewa, A. F. Tawfic, Mostafa Tarek, Nabil Abdullah Al-Sagheer, Nabil M. Nagy

**Affiliations:** 1 Civil Engineering Department, Military Technical College, Cairo, Egypt; 2 Head of Nuclear Engineering Department, Military Technical College, Cairo, Egypt; 3 Aluminum Sulfate Co. of Egypt, Cairo, Egypt

**Keywords:** Adsorption, Aluminum sulfate residue, Geopolymer, Heavy metal removal, Metakaolin, Water treatment

## Abstract

A geopolymer (GP) from Partially Dealuminated Kaolin (PDK) was synthesized. PDK is a solid waste of alum industry, it was produced in a big quantity, which need careful management to be recycled for protection of the environment against pollution. Utilization of PDK is very lacking, and there were no studies for using in the preparation of geopolymer as an adsorbent for heavy metal removal from wastewater. GP was used for the removal of Cr, Cd, and Pb from synthetic industrial wastewater by the adsorption technique. FTIR spectrum indicates a peak at 977 cm^−1^ due to Si–O–Si and Si–O–Al bonds confirming the formation of geopolymer. The effects of various parameters such as temperature, pH, contact time, and metal ion concentration were tested to stand over the most favorable conditions for adsorption. A total of 100% removal was achieved at a pH = 6.0, temperature = 25 °C, and initial concentration = 40 mg/L for a contact time of 60 min using a dosage of 0.2 g/L. The adsorption data validated Freundlich adsorption model. The values of Freundlich constant value, R^2^ were greater than 0.99 indicating the adsorption of metal ions onto the geopolymer to be highly favorable. High adsorption capacity has been achieved for Pb, Cd, and Cr (105.6 mg/g for Pb, 150 mg/g for Cd, 125 mg/g for Cr). The adsorption process followed pseudo-1st-order kinetics yielding high correlation coefficient and the adsorbed amount at equilibrium. More than 95% of adsorption was achieved at room temperature supports the effectiveness of metal ions adsorption on the geopolymer. This work helps for the reuse of the industrial waste of alum industry through the synthesis of a geopolymeric adsorbent, which can be applied successfully for removal of the Pb, Cd, and Cr ions from the polluted water.

## Introduction

The supply and quality of water have been affected by the fast urbanization and population growth in the last several decades. The unregulated release of untreated industrial wastewater causes several environmental concerns, including heavy metal pollution^1,2^. Ions of heavy metals are defined as those containing an elemental metal with an atomic weight beyond 63.5 and a specific gravity above 5.0 g/cm^3^^3,4^. Heavy metals are mobilized to a large extent by activities including mining, metal plating, petrochemical processing, paper manufacturing, and fertilizer manufacture. Copper, arsenic, mercury, nickel, cadmium, lead, and chromium are the most hazardous heavy metals, according to the US Environmental Protection Agency US EPA^5,6^. Because of their toxicity, bioaccumulation, and inability to break down in the environment, heavy metals pose a significant risk to every living thing on our planet, even at low quantities. Many devastating health problems, including cancer, cardiovascular disease, brain tumors, and neurological dysfunction, are brought on by heavy metal ion exposure to humans. Heavy metal removal from water is therefore necessary for environmental and human health protection^7–9^.

Among the several techniques used to purify wastewater, chemical precipitation stands out as an effective strategy for eliminating heavy metals from industrial effluent. Filtration is used to remove heavy metals from a solution after they have precipitated as sulfides or hydroxides as a result of a chemical reaction with reagents^10^.

Another method for removing heavy metals is membrane filtration. It employs a number of membrane technologies, including as reverse osmosis, nanofiltration, and ultrafiltration. This kind of purification relies on the molar mass of the metal and the pore diameters of the membrane^11,12^. Ion exchange, a technique that effectively removes heavy metals, has found commercial applications for both synthetic and naturally occurring ion exchange resins^13^.

Because of its versatility, low startup costs, high efficiency, and ease of use in both batch and continuous operations, adsorption stands out as the top physicochemical method for heavy metal removal^14,15^. Examples of adsorbents include activated carbon (AC), biochar (BC), clay minerals, chitosan, lignin, and geopolymer.

Over 250,000 tons were emitted by Egypt in 2023. The pH scale measures how acidic a material is when it is less than 4, PDK. It is difficult to remove due to the tiny particle size and acidic characteristics. Disposal procedures have significant impacts on the environment and need extensive storage space. Because it is mostly siliceous, PDK may be used as a component in the manufacturing of geopolymers^16–18^.

Geopolymer has garnered significant attention among academics as an adsorbent because to its superior immobilization capabilities. Geopolymer is an inorganic polymer characterized by a three-dimensional (3D) polymeric structure and the presence of pores. by the condensation of aluminosilicate mineral powder introduced into an alkali solution at temperatures below 100 °C, a process pioneered by Joseph Davidovits in 1970^19–21^. The analogous zeolite structure of geopolymer imparts superior adsorption characteristics, facilitating the extraction of heavy metals from wastewater. Geopolymer has features akin to zeolite, demonstrating a significant capacity for cation exchange and a pronounced attraction for cationic heavy metals due to the presence of Aluminum in its matrix^22–24^.

Moreover, geopolymer can be synthesized utilizing geological materials such as kaolin, metakaolin, and dolomite, as well as industrial byproducts like slag, fly ash (FA), and sludge as aluminosilicate precursors. Metakaolin (MK) is a widely utilized aluminosilicate material for the production of geopolymer-based adsorbents, as it provides distinctive adsorption characteristics, including varied structural selectivity, enhanced sorption capacity, and cation exchangeability for multiple metal cations, thereby facilitating the optimization of wastewater treatment process design^25–28^.

The alkali activation of aluminosilicate minerals, such as fly ash, granulated blast-furnace slag, and metakaolin, yields geopolymers, which are inorganic polymeric compounds. In the 1970s, Professor Joseph Davidovits first presented the term “Geopolymer” to a global audience^25,26^. The abbreviation “geo” refers to the inorganic aluminosilicate obtained from geological materials that reacted with an alkaline solution to form geopolymer via a polycondensation process^29–31^. This work seeks to employ PDK, a byproduct of the alum industry, in the fabrication of a geopolymeric adsorbent for the extraction of heavy metals, namely Pb^2+^, Cd^2+^, and Cr^6+^ ions^32,33^. The recycling of this material yields a novel source for efficient adsorbent geopolymer applicable in water treatment. It is an ecologically friendly approach for enhancing industry value.

## Experimental programm

**Fig. 1:**
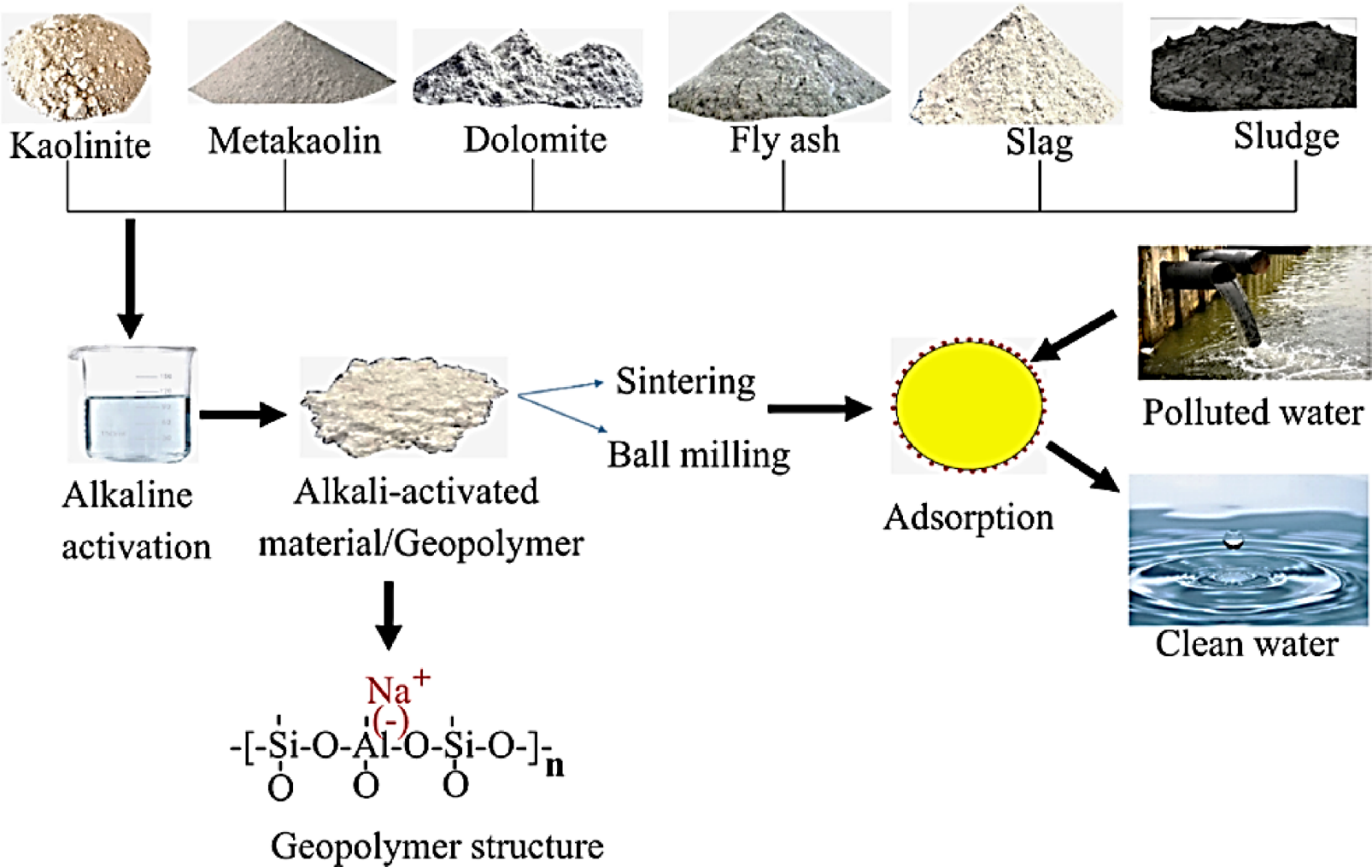
Illustration of geopolymer synthesis^29^.

The Aluminum Sulfate Company of Egypt supplied the kaolinite (Al_2_Si_2_O_5_(OH)_4_) used to make metakaolin (MK), which is rich in silica and alumina. The process of dehydration took place at 700 °C for 90 min. As an extra supply of amorphous silica, PDK also makes sure that the finished geopolymers have enough SiO_2_. When PDK and NaOH react, a powerful alkaline activator is produced. The NaOH solution was made by dissolving al-Nasr chemical company’s 99% pure NaOH pellets in distilled water. The NaOH solution had a concentration of 12 M when it was prepared. By combining PDK with a 12 M NaOH solution, an activator for alkaline silicates was created. Figure 1 shows the final product of a geopolymeric paste made by adding MK to the activator and mixing the two ingredients completely.

A solution of activator was prepared by dissolving PDK powder in 12 M NaOH and agitating it at room temperature for 15 min. The activator and metakaolin were then combined in a mixer at room temperature for three minutes while being swirled constantly to achieve an adequate degree of homogeneity. The 50 × 50 × 50 mm cubic plastic mold was used after the mixture was swiftly mixed at ambient temperature. After that, we dehydrated for a day at 70 °C. The resulting geopolymer was milled and sieved to achieve a particle size between 63 and 200 μm. After that, until the pH was adjusted, rinse it well with purified water. Compressive strength was measured to determine the mechanical properties of the geopolymer (Table 1)^34^.

**Table 1:** Geopolymer ingredients, and the mix design.

Sample	MK	SiO_2_	Al_2_O_3_	Partially de-Aluminated metakaolin	SiO_2_	Al_2_O_3_	Total SiO_2_ Total Al_2_O_3_	NaOH	Water	Average Compressive Strength	Molar ratio SiO_2_/Al_2_O_3_
				“Source of Silica”							
	(g)			(g)				(g)	(g)	(MPa)	
#1	280	116.8 1.95 M	98 0.96 M	264	167.1 2.78 M	16.4 0.16 M	4.73 1.12	140	126	21	4.2
#2	330	137.6 2.29 M	115.5 1.13 M	205	129.8 2.16 M	12.7 0.125 M	4.45 1.26	165	110	28	3.5
**#3**	**377**	**157.2** **2.62 M**	**132** **1.29**	**151**	**95.6** **1.59 M**	**9.4** **0.092**	**4.21** **1.38**	**188**	**95**	**43**	**3.05**
#4	425	177.2 2.95 M	148.8 1.46 M	94	59.5 0.99 M	5.8 0.057 M	3.94 1.52	212	80	32	2.6

M: Molar ratio.

Molar ratio = weight / molecular weight. For silica weight (g) divided by 60; For Alumina weight (g) divided by 102.

Significant values are in bold.

### Geopolymer characterisation

Geopolymer precursor and adsorbent characterization is crucial for evaluating synthesis methods and final product design. Deionized water was used to rinse the produced geopolymers in order to remove any surplus of sodium hydroxide. Grinding the materials after drying and passing them through a 63–200 mesh screen allowed us to control the particle size distribution. Prior to conducting metal ion adsorption tests, powder samples underwent analysis using FTIR spectroscopy for the detection of geopolymer molecular vibrations, X-ray diffractometry (XRD) for the analysis of crystal structures, X-ray fluorescence (XRF) for the determination of the chemical composition of the primary components, scanning electron microscopy (SEM), and Energy Dispersive X-ray spectroscopy (EDX) for the evaluation of geopolymer the structure. The surface area of the Brunauer-Emmer-Teller (BET) was measured by N2 adsorption–desorption after drying at 100 °C. A potentiometer, specifically an ELSZ1NGK Photal Otsuka Electronics instrument, was used to measure the zeta potential both with and without 5 and 10 weight percent sodium chloride.

### Adsorption procedure for heavy metal

The adsorption procedures of the geopolymers were conducted in a controlled setting with a temperature of 25 °C and a pH of 6. The bulk of the water-based solutions containing Pb^2+^, Cd^2+^, and Cr^6+^, with concentrations varying from 10 to 40 mg/L, were derived from analytically-grade standard solutions. The pH of the heavy metal solution was adjusted to 6 using 0.1 M HCl and 0.1 M NaOH before to each adsorption experiment. Fifty milliliters of the water solution containing the metal ions was then added with 0.1 mg of geopolymer powder. After the adsorption experiment, the supernatant was separated by centrifugation at 100 rpm. The AA Spectrometer—6300 SHIMADZU was used to analyze the supernatant’s concentration changes of metal ions. Several variables were taken into account, including measurements of lead, cadmium, and chromium ions, contact duration, and water pH. The concentration of metal ions in the solution changed as a result of geopolymer adsorption, as shown in the following equation.

1 $${\text{Q}}_{{\text{e}}} = \, \left( {{\text{C}}_{{\text{o}}} {-}{\text{ C}}_{{\text{e}}} } \right){\text{ V}}/{\text{W}}$$

The initial and equilibrium concentrations of the metal ions in the solution are denoted by Co and Ce, in parts per million (ppm), respectively. V represents the volume (L) and W the weight (mg) of the adsorbent.

## Results and discussion

In this section the characterization and the adsorption capabilities of the prepared geopolymer to eliminate the selected heavy metals from the industrial wastewater are demonstrated.

### The PDK characterization

**Fig. 2:**
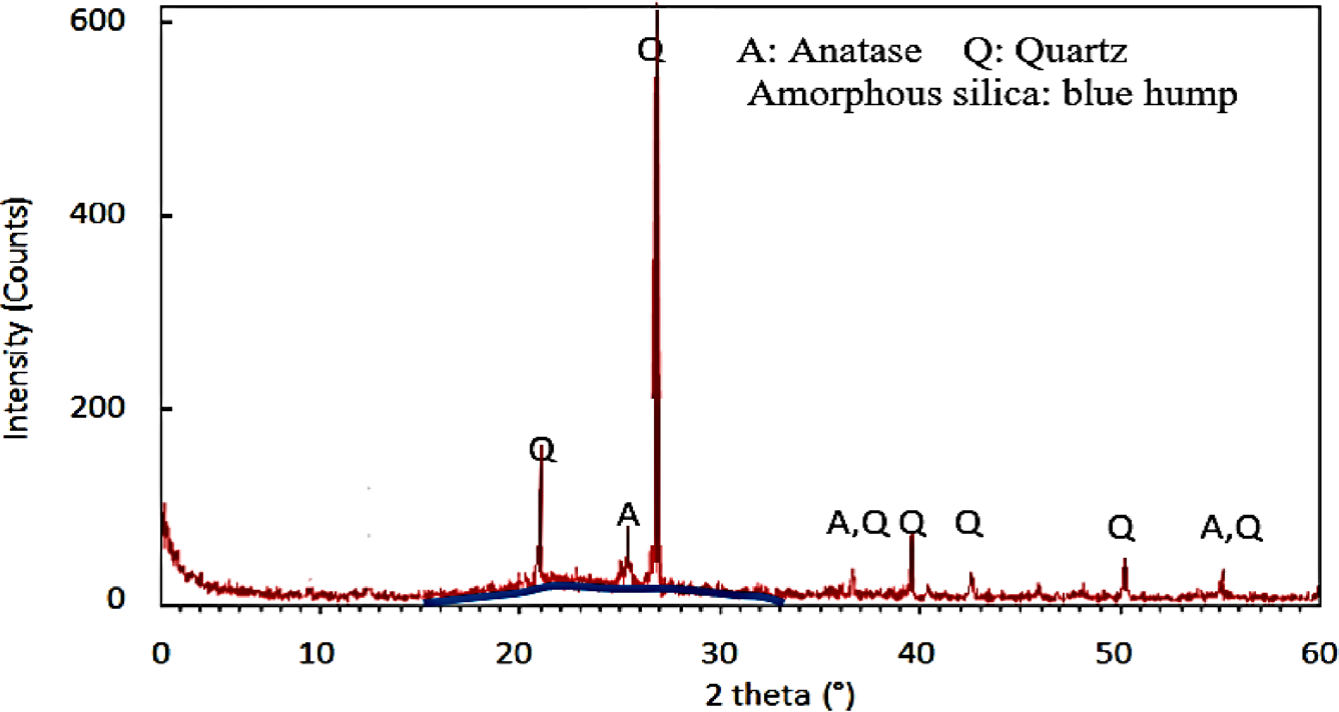
PDK X-ray Diffraction.

The undersized diameter of PDK at 90% of the sample is around 75 microns, at 10% it is around 5.0 microns, with a mean diameter of approximately 10 microns. The BET surface area of PDK is 20 m^2^/g, determined by the Brunauer, Emmett, and Teller (BET) technique. Table 2 indicates that the chemical composition of PDK reveals amorphous SiO_2_ as the predominant component of the residue, constituting up to 62.3%, while crystalline silica comprises 18.2% of PDK. Besides iron oxides, PDK comprises many leachable elements: Ti, Fe, and Al. The crystalline phases of PDK may be categorized into two types: (1) Silicates: quartz (SiO_2_); (2) Anatase (TiO_2_), as evidenced by the XRD (Fig. 2).

**Table 2:** the XRF shows the chemical composition of PDK.

Parameter	SiO_2(qz)_	SiO_2(am)_	Al_2_O_3_	Fe_2_O_3_	TiO_2_	SO_3_	Loss of ignition	Total
Value (%)	18.2	62.3	7.32	0.55	3.35	1.3	6.7	99.72

**Fig. 3:**
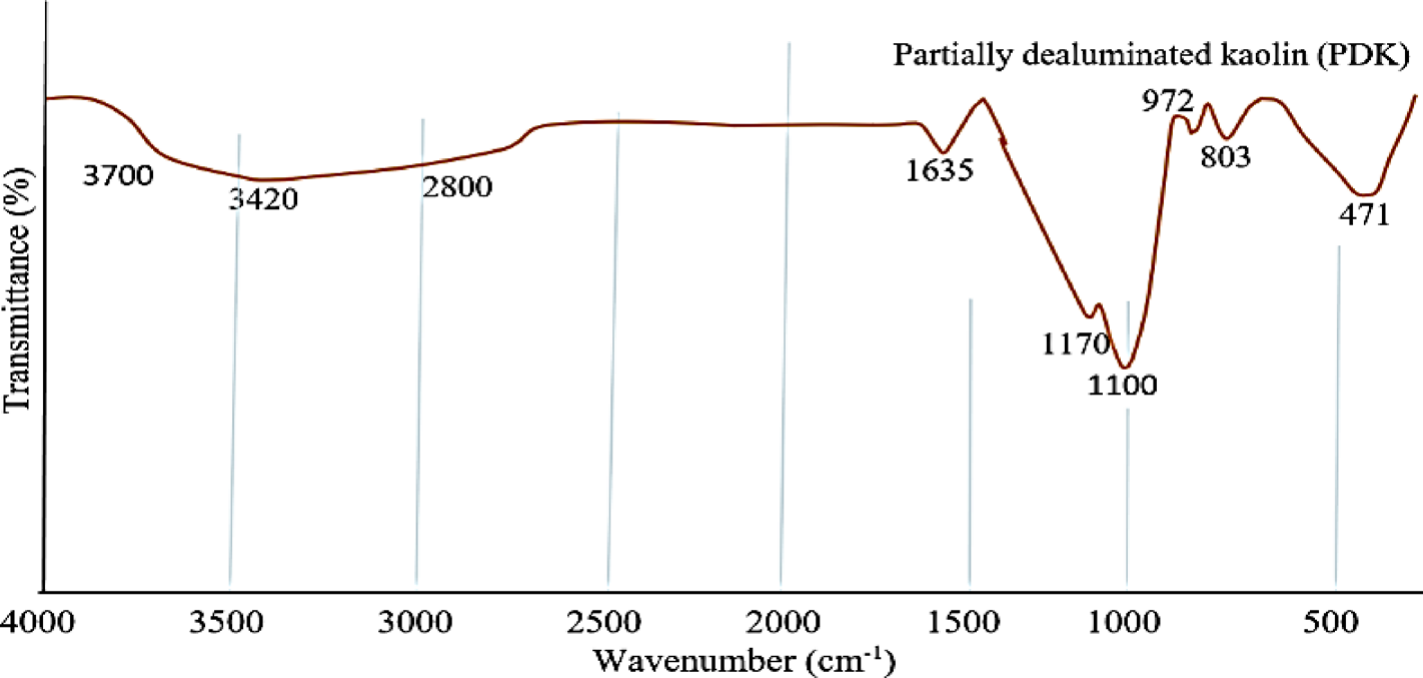
FTIR spectrum for PDK.

Figure 3 shows the FTIR spectrum for PDK. The spectrum shows three bands at 1100, 803, and 471 cm^−1^, which indicates a lower degree of polymerization of the silica network. The frequencies of the Si–O–Si bands are an indication for the overall degree of polymerization of the silica network. PDK spectrum also shows a band at 1170 cm^−1^, due to the presence of sulfate ion, and at 972 cm^−1^ due to the Si–O–Si stretching siloxane bond. The band at 1635 cm^−1^ is due to the H–O–H bending vibration of molecular H_2_O indicating a water absorption), and the broad band at 2800–3700 cm^−1^ is due to the stretching vibration of –OH groups in H_2_O hydroxyls with a wide range of hydrogen bonding strengths^35–38^. The absorption bands account for noticeable large loss of ignition (LOI) of PDK, which results from the loss of hydrogen bonded water (Si–OH:OH_2_) and pyrolysis of silanol (Si–OH) groups, formed during acid leaching of calcined kaolin (metakaolin). The vibration signal at 1100 cm^−1^ was the asymmetric stretching of Si–O–Si. Peak at 803 cm^−1^ represented the symmetric stretching of Si–O–Si bond^39–43^. The peak at 471 cm^−1^ was assigned to the asymmetric bending of Si–O–Si bond^44^.

**Fig. 4:**
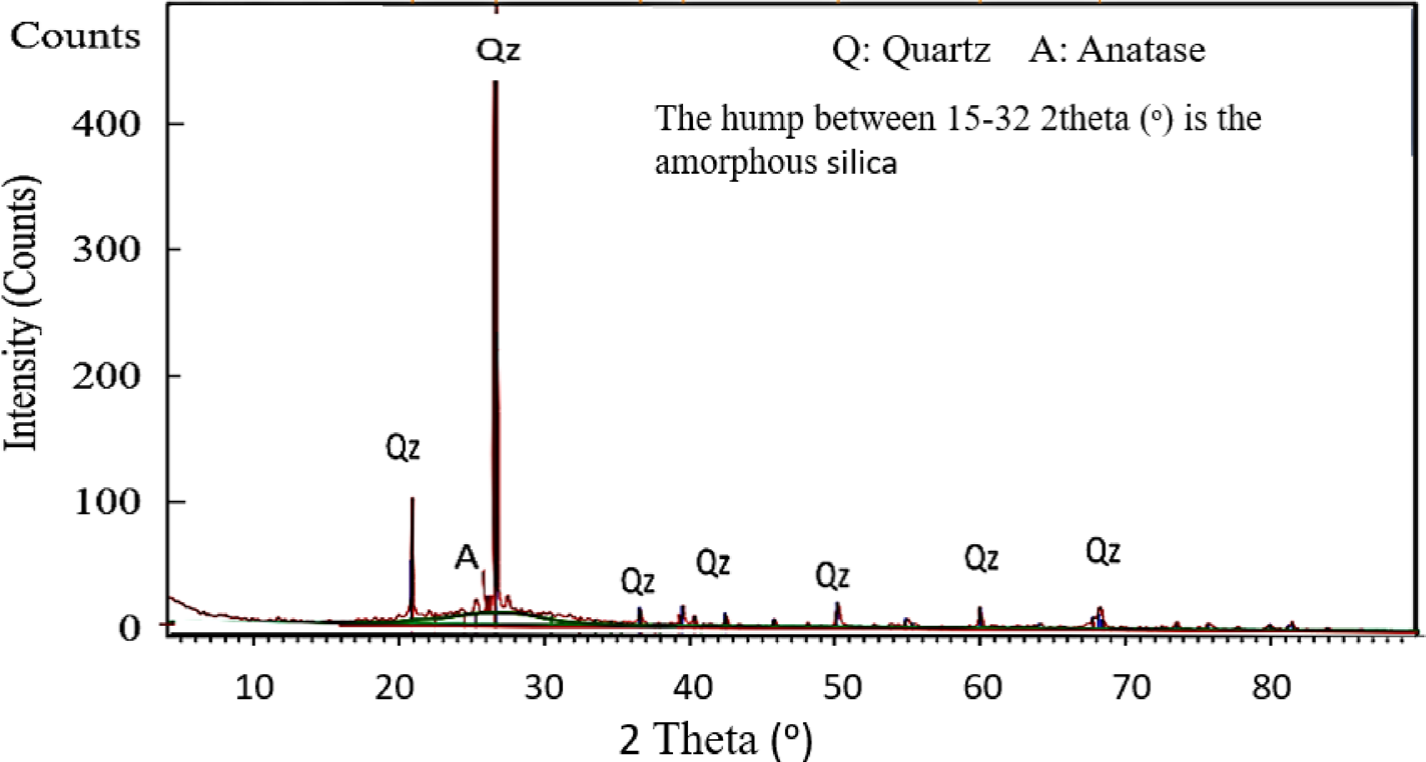
X-ray Diffraction of Geopolymers.

In Fig. 4, XRD The analysis of the amorphous phase of the geopolymer demonstrates the successful creation of the geopolymer matrix, as this disordered structure is characteristic of geopolymers. The detected quartz presumably derives from metakaolin or other additives and stays partially unreacted during the geopolymerization process, frequently functioning inertly within these systems. The existence of an anatase phase can be ascribed to the incorporation of TiO_2_ in the initial materials or as an addition. X-ray diffraction (XRD) examination of the geopolymer identifies unreacted crystalline phases, including anatase and quartz, in conjunction with an amorphous matrix defined by a wide hump. This mostly amorphous form is characteristic of geopolymers; nevertheless, crystalline components also indicate the presence of unreacted elements or additions inside the geopolymer (Fig. 4).

#### FTIR Analysis for prepared geopolymer

**Fig. 5:**
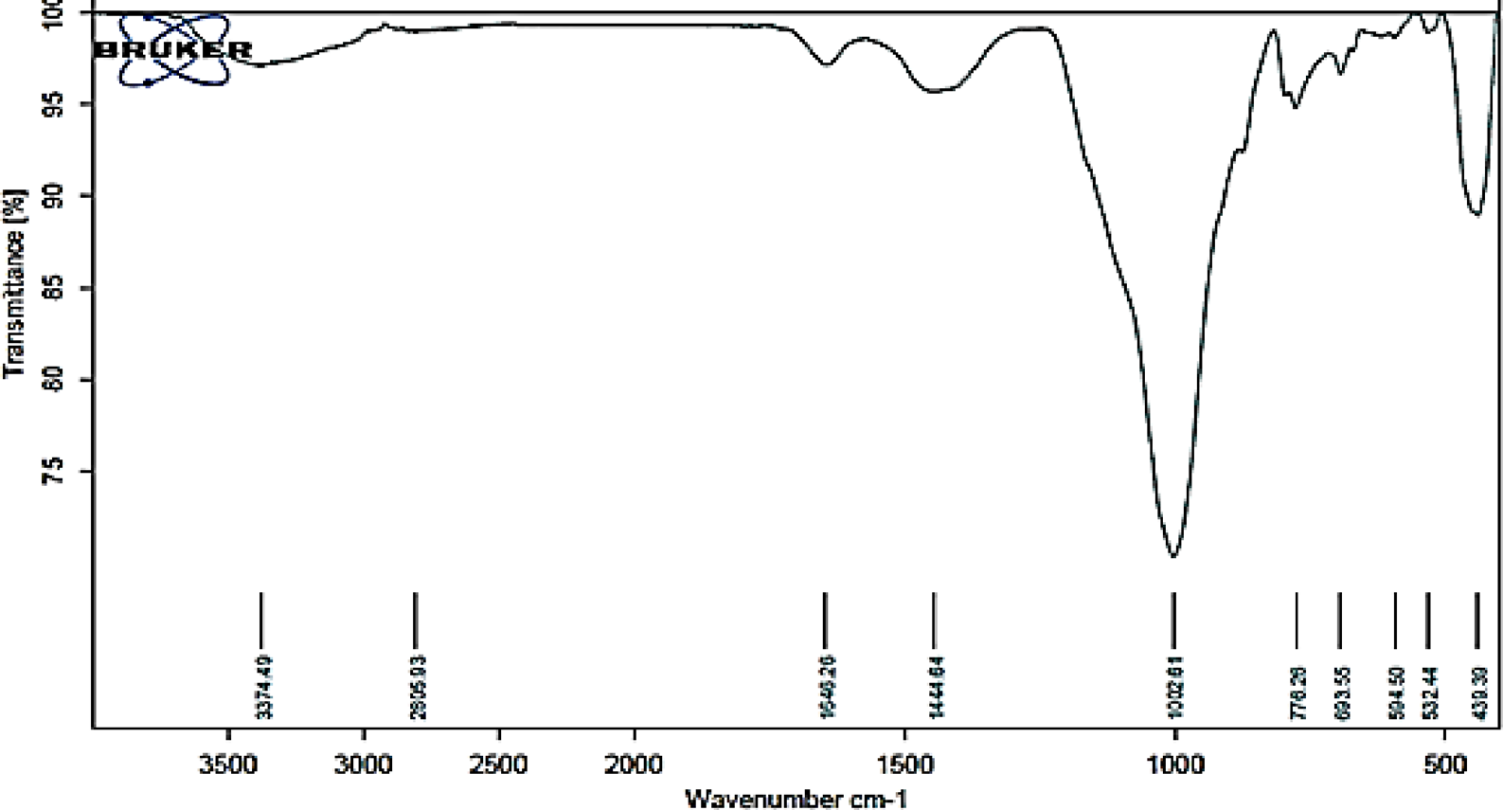
FTIR of a Geopolymer.

The FTIR spectra of the synthesized geopolymer blends with varying ratios of PDK are shown in Fig. 5. A broad band of O–H stretching and bending is seen in the region of 3435–3457 cm^−1^ in the geopolymers, indicative of hydrogen-bonded surface OH groups, including the aluminol and silanol groups. The bending vibration of H–O–H, included within the aluminosilicate framework, was attributed to the band seen at 1647.5 cm^−1^. A prominent distinctive band of Si–O was found at 977 cm^−1^, indicating asymmetric stretching vibrations of [Si–O–Si] or [Al–O–Si]^45–48^. Moreover, the symmetric stretching vibrations (Si–O–Si) at 770 cm^−1^ and 690 cm^−1^, as well as the bending vibrations (Si–O–Si) and (O–Si–O) about 500 cm^−1^, exhibit considerably greater band intensities in the M17 sample compared to the other samples. The Si–O–Si bond vibration is denoted by the wavenumber 685.8 cm^−1^^49–51^.

#### XRF of geopolymer

A synthetic geopolymer was discovered to have enhanced compressive strength. Table 3 shows the main elements for geopolymer manufacturing according to the XRF study: SiO_2_, Al_2_O_3_, and Na_2_O.

**Table 3:** XRF of the prepared geopolymer.

The parameters (%)									
SiO_2_	Al_2_O_3_	Fe_2_O_3_	TiO_2_	Na_2_O	CaO	P_2_O_5_	Cl	Loss of ignition	Total
55.7	20.7	1.44	2.14	7.6	0.32	0.09	0.03	16.3	99.33

#### SEM and EDX

**Fig. 6:**
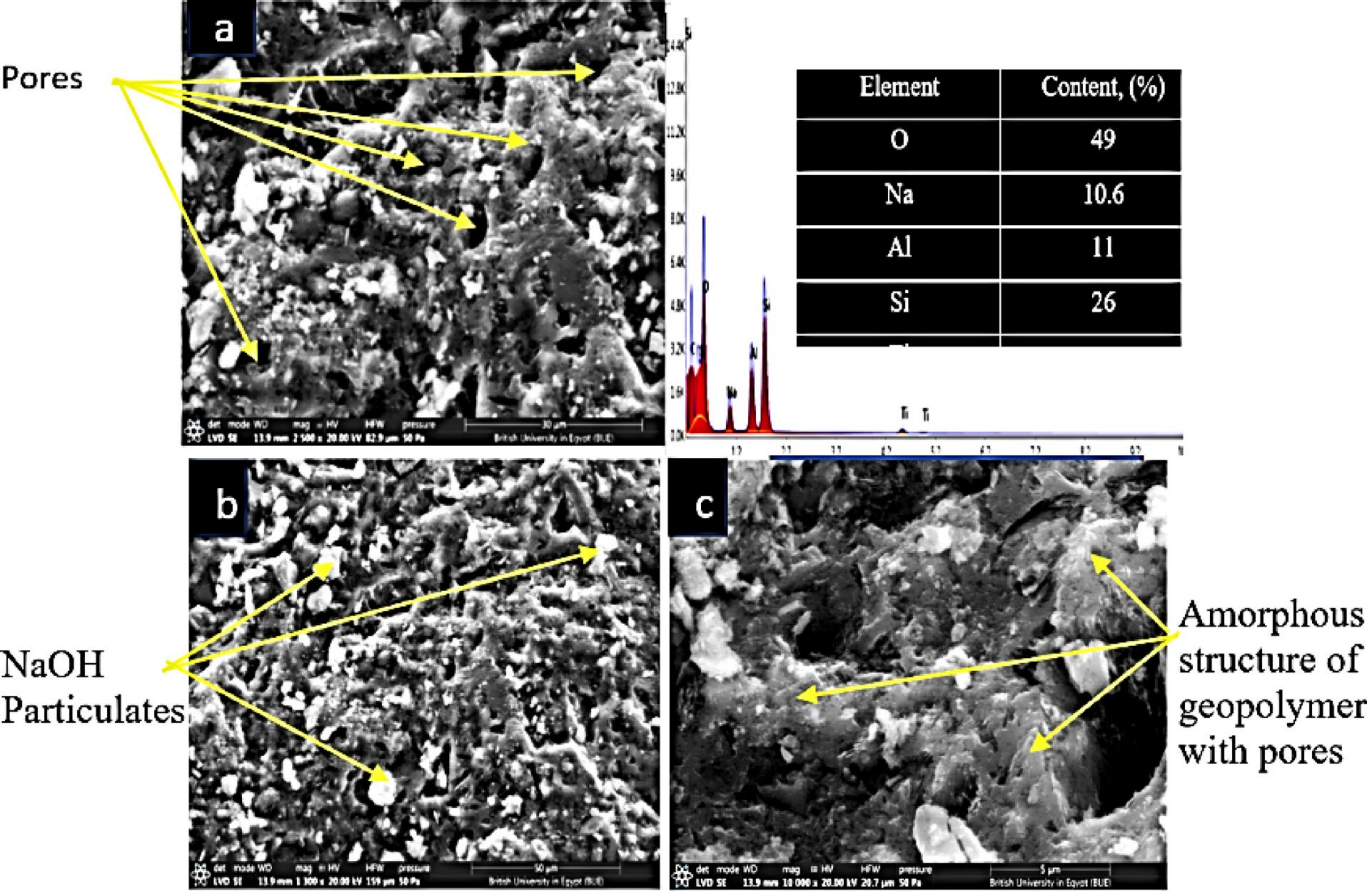
Displays EDX analysis with geopolymer SEM images (a, b, c) taken at several magnifications.

Figure 6 displays the surface morphology of a geopolymer particle at a magnification of 500×. Grainy and gritty, the surface is similar to geopolymers made from metakaolin. Rough surfaces allow pollutants, including heavy metals, to be adsorbed onto a wider surface area. It seems that particles or clusters inside the designated region have non-standard shapes. Metakaolin and other aluminosilicate minerals form geopolymer networks that display this peculiarity when they polycondensation in an acidic environment. Any presence of surface particles or clusters, regardless of size, indicates heterogeneous geopolymerization. Unreacted metakaolin or extra geopolymerization processes might be among these minuscule particles. By using EDX microanalysis, the elemental composition of geopolymer and metakaolin was determined. The EDX findings showed that 49% was oxygen, 10.6% was sodium, 11% was aluminum, and 26% was silicon. The XRF analysis confirmed that the produced geopolymer had the expected amounts of silicon and aluminum.

### Factors affecting the removal of Cd, Pb, and Cr

In this section the factors affecting the removal of the selected heavy meats will be presented as follow the effect of initial concentration for heavy metals, the effect of geopolymer dose on adsorption and effect of pH on adsorption.

#### Effect of the initial concentration

**Fig. 7:**
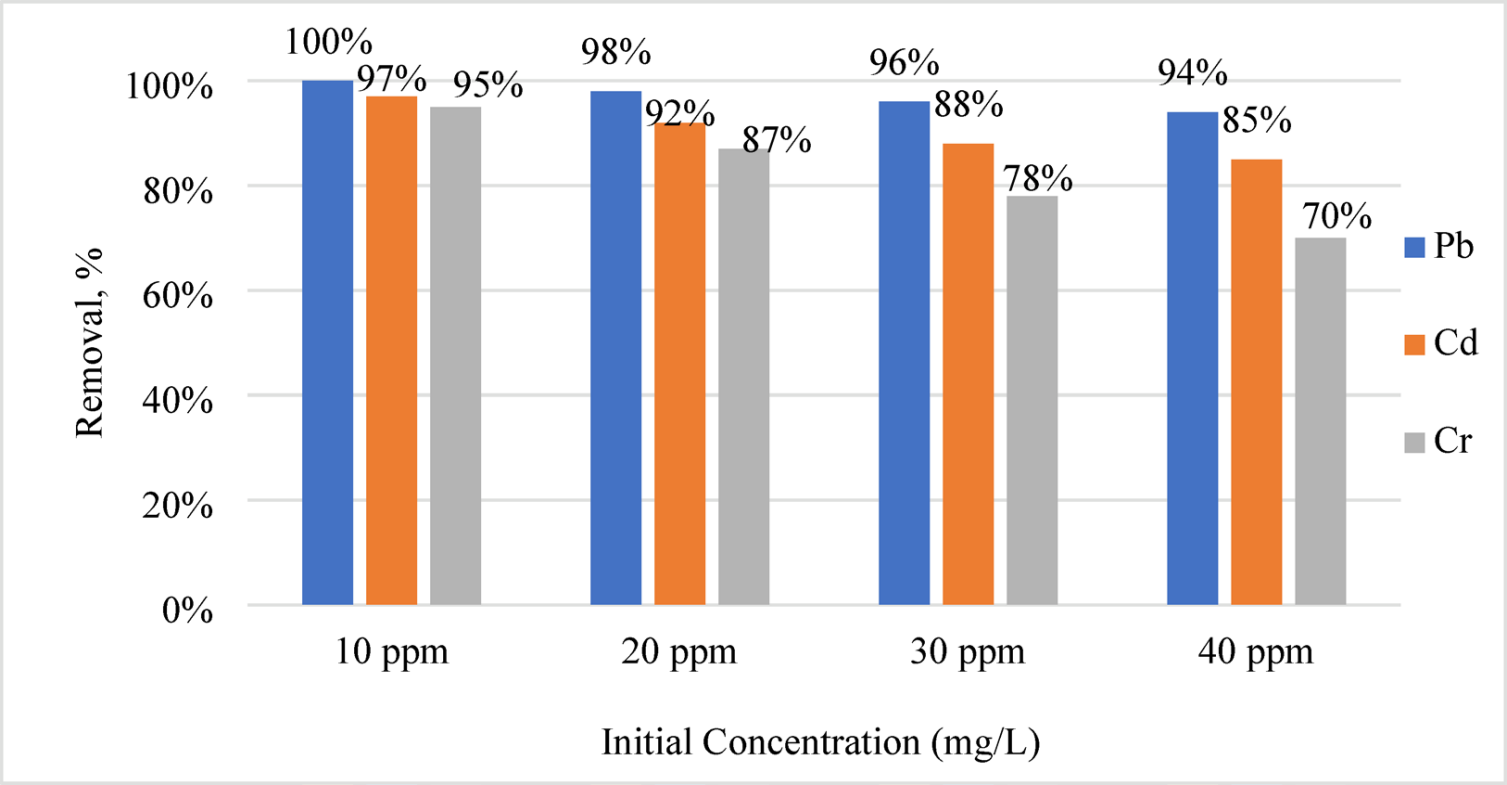
The breakdown of lead, cadmium, and chromium at different initial concentrations.

By varying the quantities of Pb, Cd, and Cr ions from 10 to 40 ppm at room temperature, we were able to examine the impact of various starting concentrations on geopolymer adsorption under ideal conditions. Figure 7 displays the results. At low concentrations, metal ions bind rapidly to the adsorbent by ion exchange or ionic interactions because there are many adsorption sites^21,52,53^. When all binding sites are full, adsorption stops. The adsorption % decreased linearly with increasing metal ion concentration. A clearance effectiveness of almost 95% is achieved when initial concentrations are below 20 mg/L. The adsorption effectiveness decreases from 100% at 10 mg/L to around 94% at 40 mg/L, indicating that metal ion absorption decreases with increasing starting concentration, even with a constant adsorbent dose of 0.03 g/L. Residual free energy changes when adsorbed onto a solid surface because of this^54^. The free energy required for adsorption often decreases as the process advances. As the concentration of the adsorbed layer increases, its reactivity towards the metal ions in solution also increases^55–57^. The pores on the surface of the geopolymer may also become too tiny due to competitive interactions, making it unable to capture all metal ions in concentrated forms^58^. As metal ion concentrations increase, the adsorbed layer’s metal ions attempt to migrate to the bulk phase, reducing adsorption. The correlation between concentrations of metal ions and adsorption densities follows consistent patterns throughout the literature^59–61^.

#### Effect of dosage of geopolymer on adsorption of Cd, Pb, and Cr

**Fig. 8:**
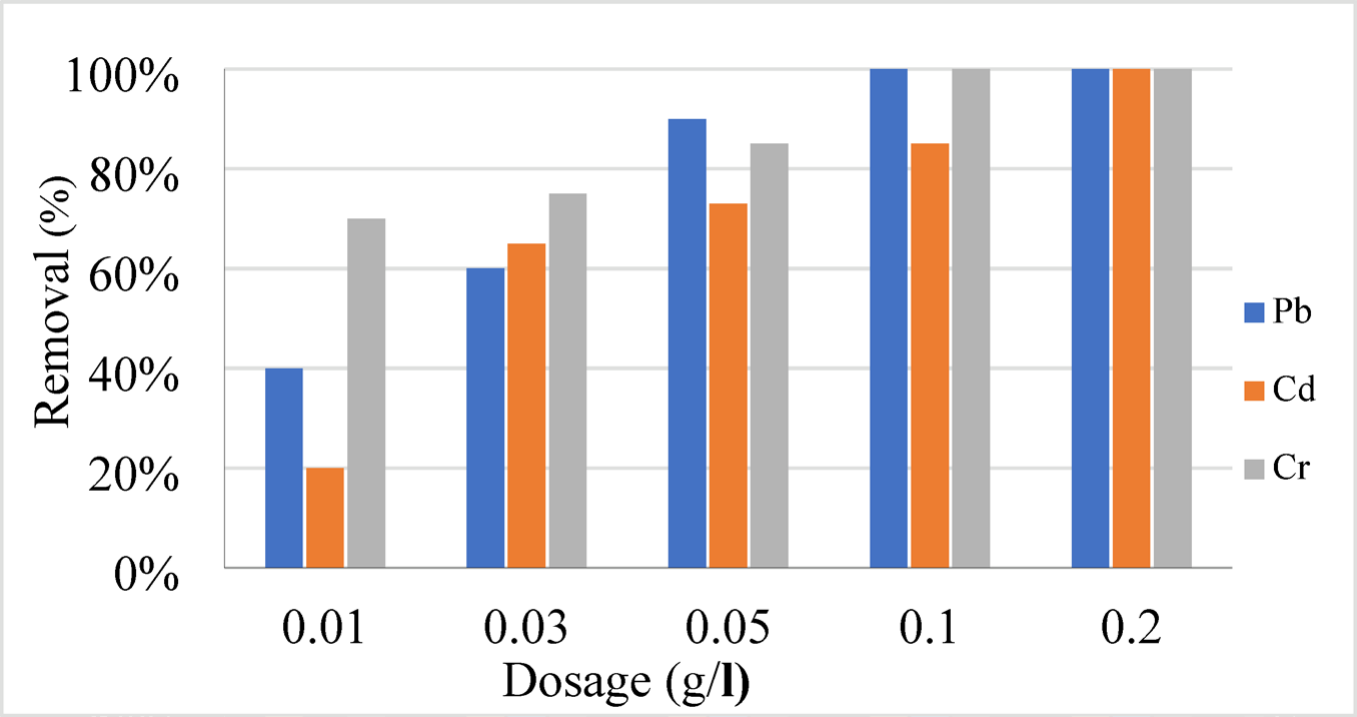
The elimination of Pb, Cd, and Cr at a pH of 6 with different doses of geopolymer.

Starting with 40 mg/L of metal ions and contacting them for 60 min at room temperature (25 °C) and a pH of 6.0, the adsorption on the adsorbent was examined by changing the dosage from 0.01 to 0.2 g/L. The outcome is shown in Fig. 8. There is a 40–100% improvement in elimination efficacy with a dose increase from 0.01 to 0.2 g/L. Because there aren’t enough active sites, the adsorbent can’t soak up all the ions in the solution, even at low concentrations. Due to the increased accessibility of the geopolymer surface, the removal rate grows exponentially with increasing adsorbent dose^62^. At the peak of adsorption at 0.2 g/L, all of the available active sites were used up^63^. Both the adsorbed ions on the geopolymer’s surface and the bulk metal ions stay in equilibrium.

Figure 8 shows that full elimination occurred at 100% when the dosage of the geopolymer was 40 mg/L of Cd, Pb, and Cr ions. Heavy metal ion adsorption, on the other hand, requires a higher concentration of the alternative adsorbents^59^. Between 10 and 200 mg/L of geopolymer was determined to be the optimal concentration for elimination. Because geopolymer was just as effective as other methods in removing lead, cadmium, and chromium, even at very high concentrations, the results lend credence to the employment of co-adsorption and co-precipitation techniques.

#### Effect of temperature on the adsorption

**Fig. 9:**
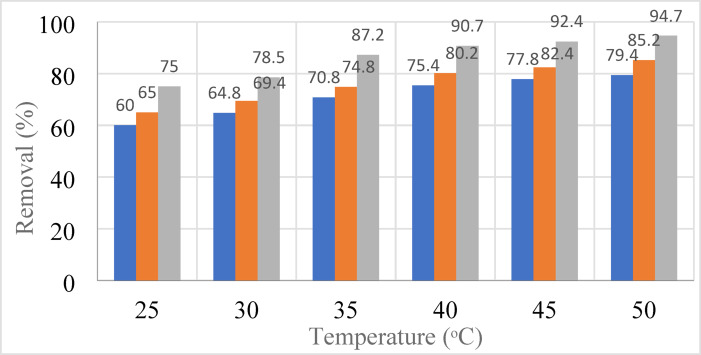
Effect of temperature on the adsorption process using 0.03 g/L, pH = 6, for 60 min.

Figure 9 shows the effect of temperature on the adsorption process. It is found that heating has a positive impact on the capacity of adsorption, confirming that the adsorption proceeds with endothermic process supporting the chemical adsorption. Also, thermal energy activates the transfer of Pb, Cd, and Cr from the liquid state to the solid state. The effect of increasing of temperature is not significate on the adsorption process, therefore it was better from the economic point of to perform the experiments at the ambient the was 25 °C with changing the other factors such as pH and dosages.

#### Effect of pH on adsorption of Cd, Pb, and Cr

**Fig. 10:**
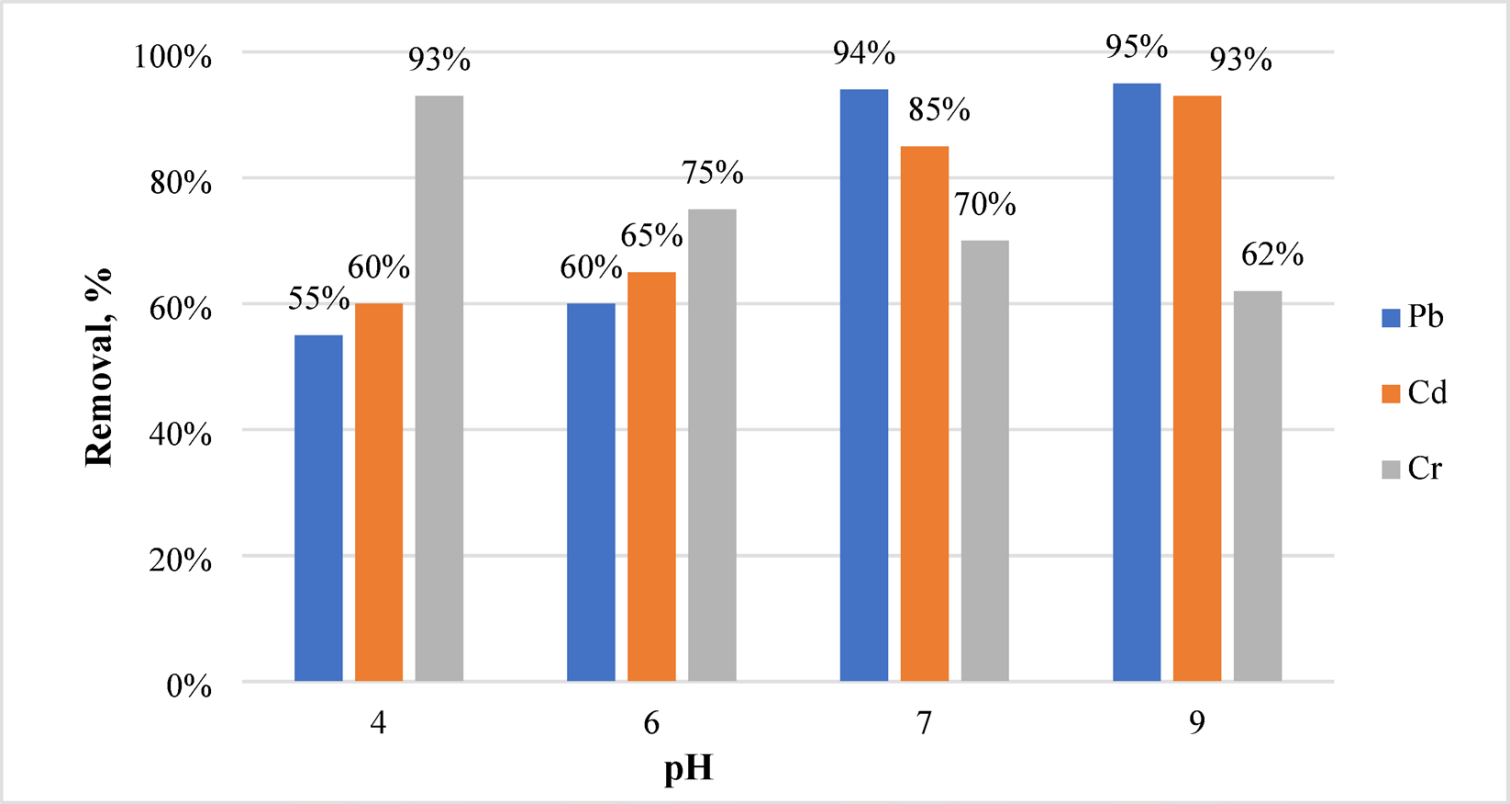
Removal Pb, Cd, and Cr at different pH using 0.03 g /L.

In Fig. 10, it is essential to examine the adsorbate adsorption on geopolymer in relation to pH as this variable influences the adsorbent surface charge^58^. The excess water leachable Na_2_O was removed during washing to eliminate its effect on the pH of adsorption process. The properties of the adsorbate ions determine the optimal pH at which adsorption may occur. Using a geopolymer dose of 0.03 g/L throughout a pH range of 4–9, the effect of solution pH on the elimination of Cd, Pb, and Cr was investigated at room temperature (Fig. 10). By adding 1 M HNO3 or 1 M NaOH, the solutions were brought to a pH of 9, which enhanced the removal of metal ions to 95% for Pb and 93% for Cd. The solutions already included 40 ppm of Pb, Cr, and Cd ions. At a pH of 9, lead and cadmium work best attributing to the predominance of precipitation rather than adsorption^58^. For chromium removal, a pH of 4 is excellent, since it is 93% efficient.

Every metal has a similar tendency to be eliminated, with the most obvious increase occurring at higher pH values. The highest increase in Pb and Cd ion adsorption rates, possibly due to ion exchange, co-precipitation, or a combination of the two, was seen in the geopolymer between pH 7.0 and 9.0. When the pH of a solution is low, the geopolymer surface shows less adsorption because the positively charged metal ions (Pb^2+^, and Cd^2+^) due to competition from high concentration of hydronium ion (H_3_O^+^). However, metal ions are more likely to migrate to the geopolymer surface for adsorption when the pH is high because the concentration of H_3_O^+^ falls^64^, in addition, the heavy metals are adsorbed on the negatively charged sites of the aluminum silicate surface of geopolymer, facilitated by the functional groups, such as aluminol (–Al–OH), and silanol (–Si–OH) indicated in FTIR (Fig. 4)^65^.

#### Effect of contact time on removal

**Fig. 11:**
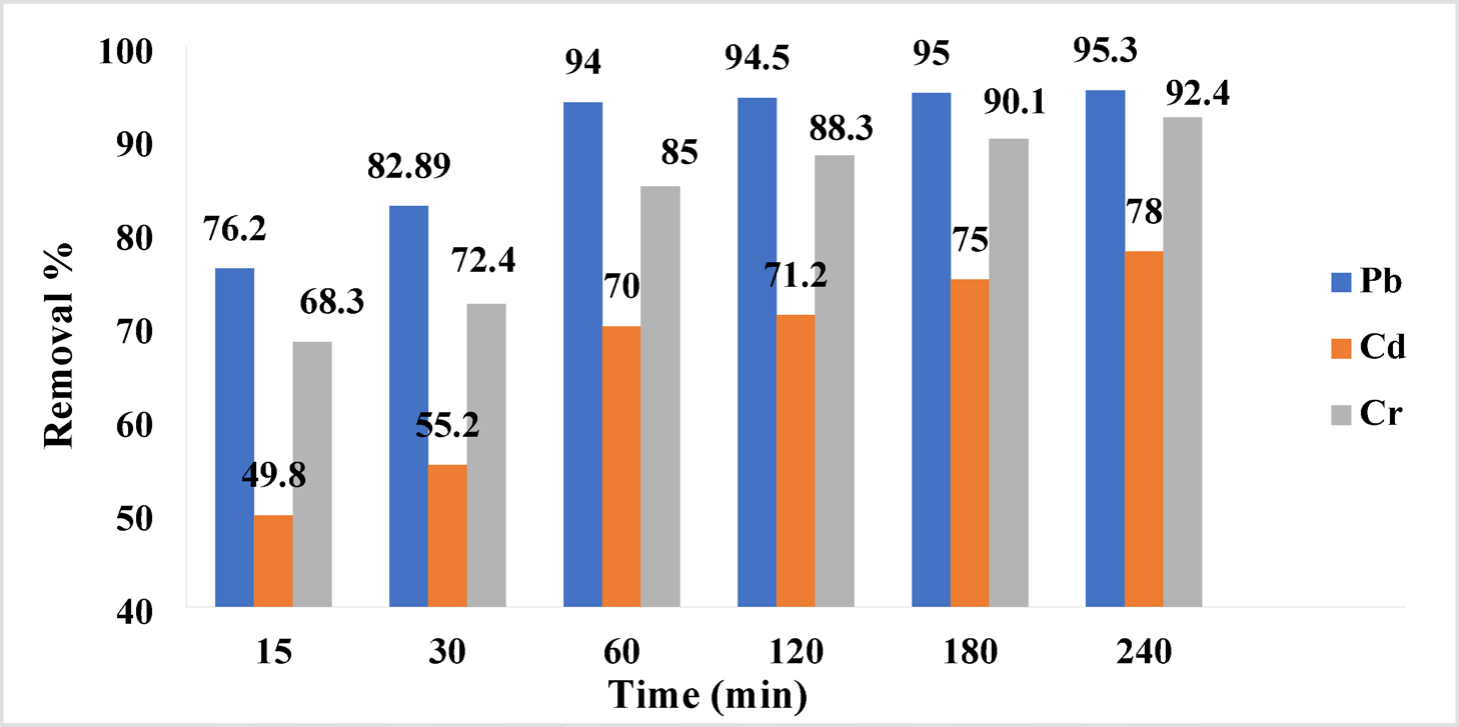
the effect of time on the adsorption.

The effect of contact time on the removal efficiency of selected heavy metals by the synthesized geopolymer is depicted in Fig. 11. The findings demonstrate that the removal percentages of Pb, Cd, and Cr increase rapidly during the initial 60 min of contact. Thereafter, equilibrium is attained within 240 min, beyond which no substantial changes in heavy metal removal are observed.

#### Adsorption isotherm

Adsorption isotherms, which are interaction profiles, may help shed light on the adsorbent-adsorbate link. The most common mathematical models created by Freundlich, Temkin, and Langmuir were used to analyze feature adsorption data, which led to the discovery of these features. These adsorption isotherms have the following assertions examined^54,55^. The Langmuir isotherm states that at a limited number of comparable locations on the adsorbent; a monolayer of adsorbate may be considered to have formed. Assumingly, there is no penetration, the adsorption surface shows uniform adsorption energy. The adsorption data is defined by C_e_, the equilibrium concentration, and q_e_, the adsorption capacity, given in mg/g. q_e_ shows the amount adsorbed at equilibrium^66^.

2 $${C}_{e}/{q}_{e}=\raisebox{1ex}{$1$}\!\left/ \!\raisebox{-1ex}{$b{q}_{0}$}\right.+\raisebox{1ex}{${C}_{e}$}\!\left/ \!\raisebox{-1ex}{${q}_{0}$}\right.$$

3 $$\raisebox{1ex}{$1$}\!\left/ \!\raisebox{-1ex}{${q}_{0}$}\right.=1/{q}_{0}+\frac{1}{b{q}_{0}}\frac{1}{{C}_{e}}$$

**Fig. 12:**
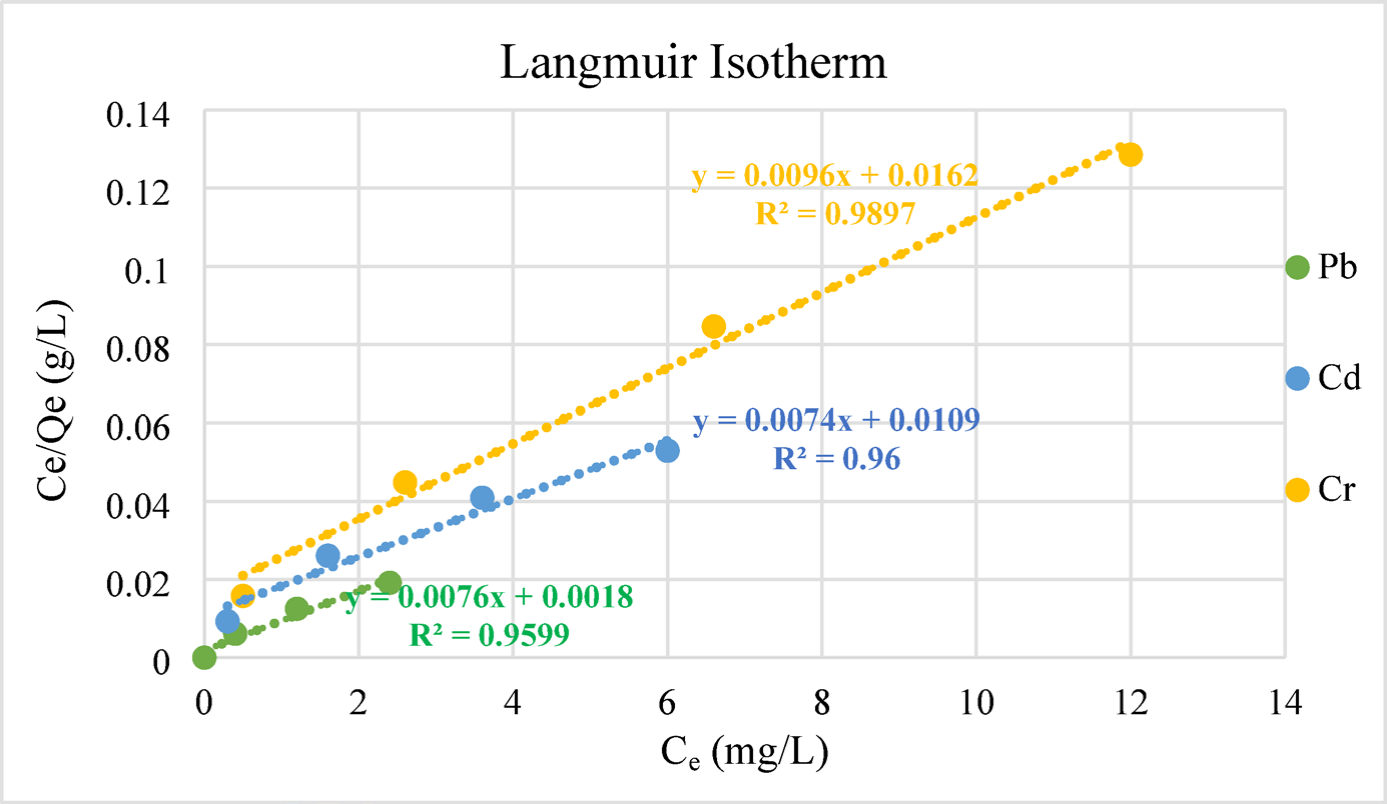
Langmuir isotherms for the removal of a Cd, Cr, and Pb by geopolymeric at 25 °C using a dosage of 0.03 g/L for 60 min, at a pH = 6.

If we plot 1/q_e_ vs 1/C_e_, where C_e_ is the equilibrium concentration in mg/L and q_e_ is the amount adsorbed at equilibrium in mg/g, we may get the Langmuir constants q_o_ and b, which are related to adsorption capacity and energy, respectively. For lead (105.6 mg/g), cadmium (150 mg/g), and crumbly metal (125 mg/g), the adsorption capacities q_o_ are shown in Fig. 12. A dimensionless constant separation factor R_L_, was found by using Eq. (4). According to references^55,57^, there are four cases based on the R_L_ number, you may determine which are: the adsorption is favorable (R_L_ < 1), unfavorable (R_L_ > 1), linear (R_L_ = 1), or irreversible (R_L_ = 0).

4 $${R}_{L}=1/(1+b{C}_{0})$$

**Fig. 13:**
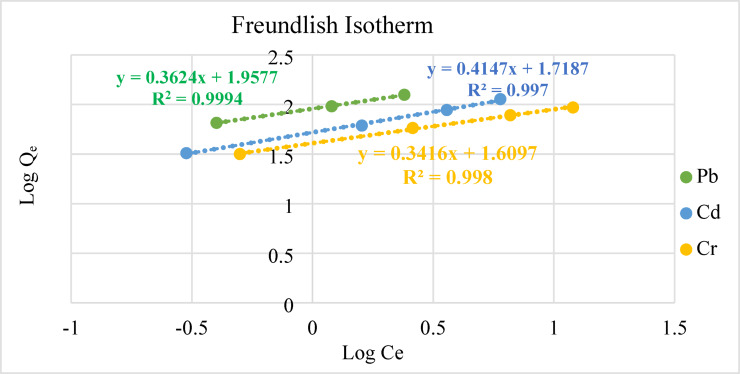
Freundlich isotherms for the removal of a Cd, Cr, and Pb by geopolymeric at 25 °C using a dosage of 0.03 g/L for 60 min, at a pH = 6.

An empirical link between the adsorbate and adsorbent is shown by the Freundlich adsorption isotherm, which, according to Eq. (5), demonstrates a linear correlation between Log C_e_ and Log q_e_. Figure 13 shows that the slope and intercept of the linear graph, where K_F_ is the adsorption capacity and 1n is the intensity of adsorption, are used to determine the Freundlich adsorption parameters.

5 $$Log {q}_{e}=\text{log}{K}_{f}+1/n\text{log}C$$

The formation of the adsorbed layer and the fluctuations in the heat of adsorption are shown in Eq. (6) of the Temkin model. Equation 7 is generated by rearranging the elements in Eq. (6). According to Eq. (7), the heat of sorption (b) is the Temkin constant in J/mol, and the Temkin isotherm constant (A_T_) is in L/g. B is therefore equal to R_T_ divided by b. The absolute temperature is denoted by T in Kelvin, but the universal gas constant is represented by R (8.314 J/mol.K).

6 $${q}_{e}\left(\frac{RT}{B}\right)\text{ln}{A}_{T}{C}_{e}$$

7 $${q}_{e}=B \text{ln}{A}_{T}+\text{ln}{C}_{e}$$

The values of A_T_ and b are obtained by plotting qe versus ln C_e_ in a linear fashion. Consistent with the previously established models, the experimentally computed characteristic parameters were also specified.

**Fig. 14:**
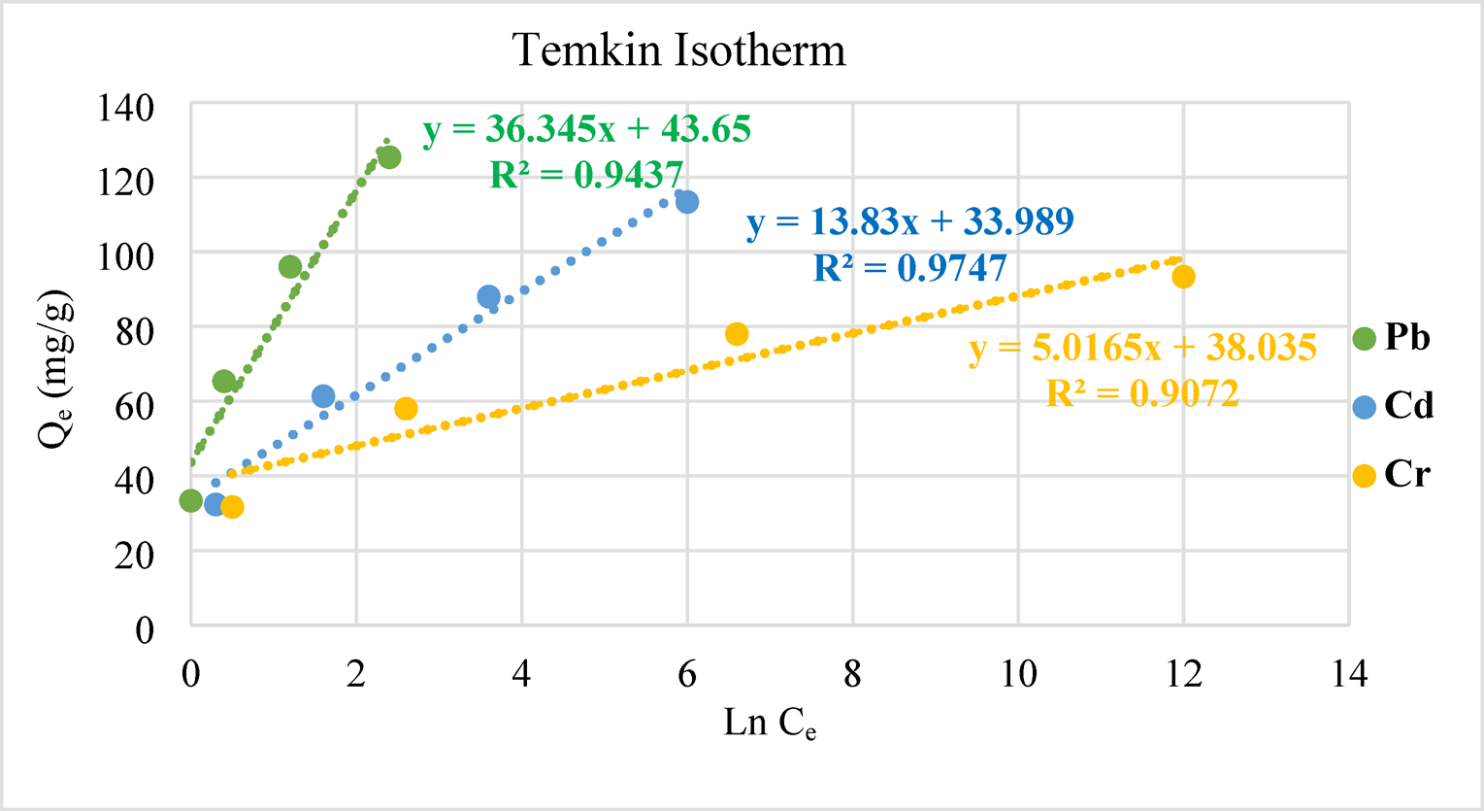
Temkin isotherms for the removal of a Cd, Cr, and Pb by geopolymeric at 25 °C using a dosage of 0.03 g/L for 60 min, at a pH = 6.

The theoretical computations of the Freundlich isotherm model shows multilayer adsorption when the linear correlations are large (R^2^ > 0.99). With a R^2^ value higher than 0.97, the Temkin isotherm models worked well for Cd adsorption, as shown in Fig. 14. However, there was insufficient Pb and Cr adsorption captured by the adsorption assays for them to be considered significant.

#### Adsorption kinetics

To assess the adsorption kinetics, the adsorption process was conducted continuously at 25ºC with a starting concentration of 40 ppm of metal ions, 30 mg of adsorbent, and a duration of one hour. Using pseudo-second-order (Eq. 9) and pseudo-first-order (Eq. 8) adsorption models, the data was evaluated, according to Naseem et al.^48^.

8 $$\text{log}\left({q}_{e}-{q}_{t}\right)=\text{log}{q}_{t}-\frac{{k}_{1}}{2.303t}$$

9 $$\frac{t}{{q}_{t}}=\frac{1}{{k}_{2}{q}_{{e}^{2}}}+1/{q}_{e}t$$

**Fig. 15:**
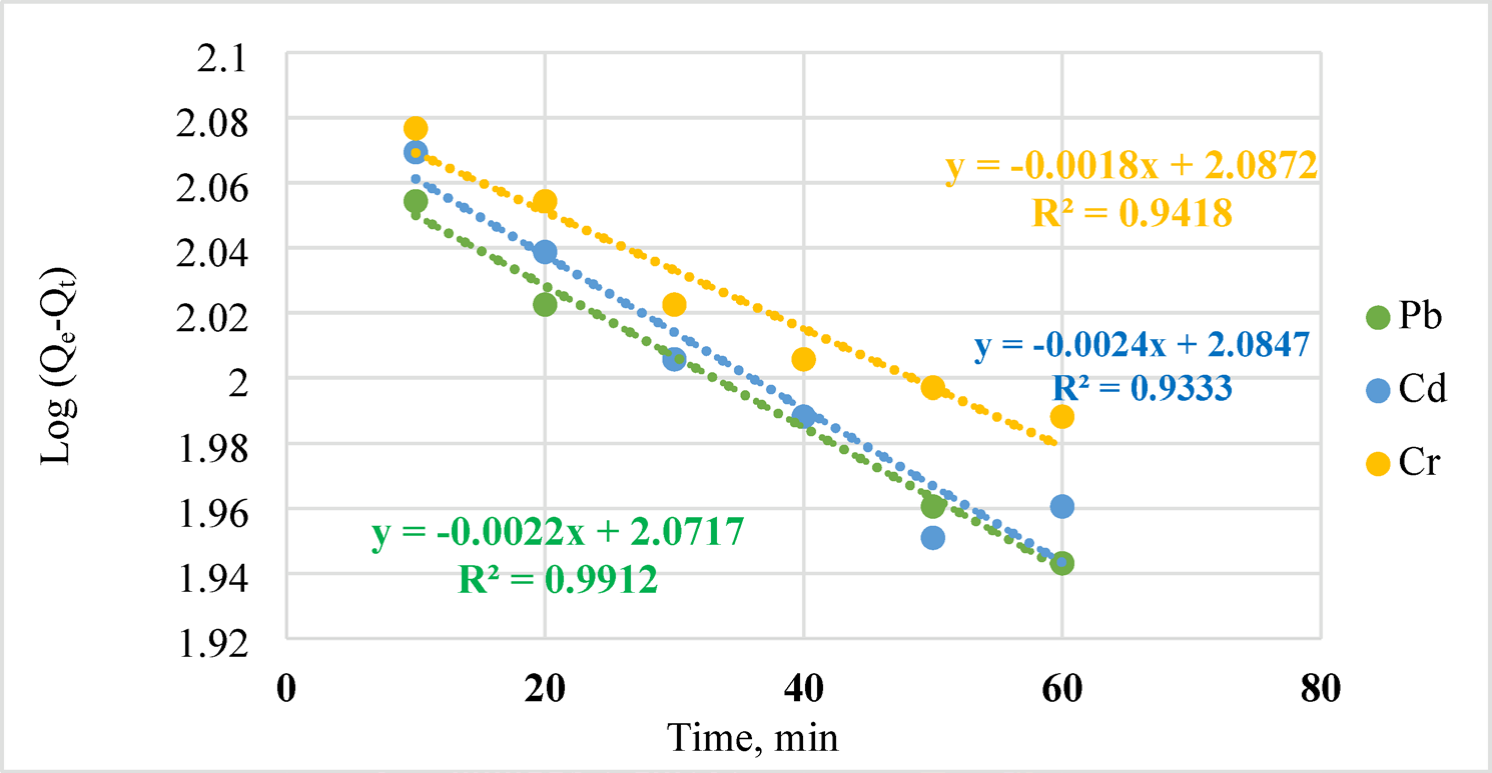
Analytical Models for Pseudo-First Order Absorption Kinetics.

**Fig. 16:**
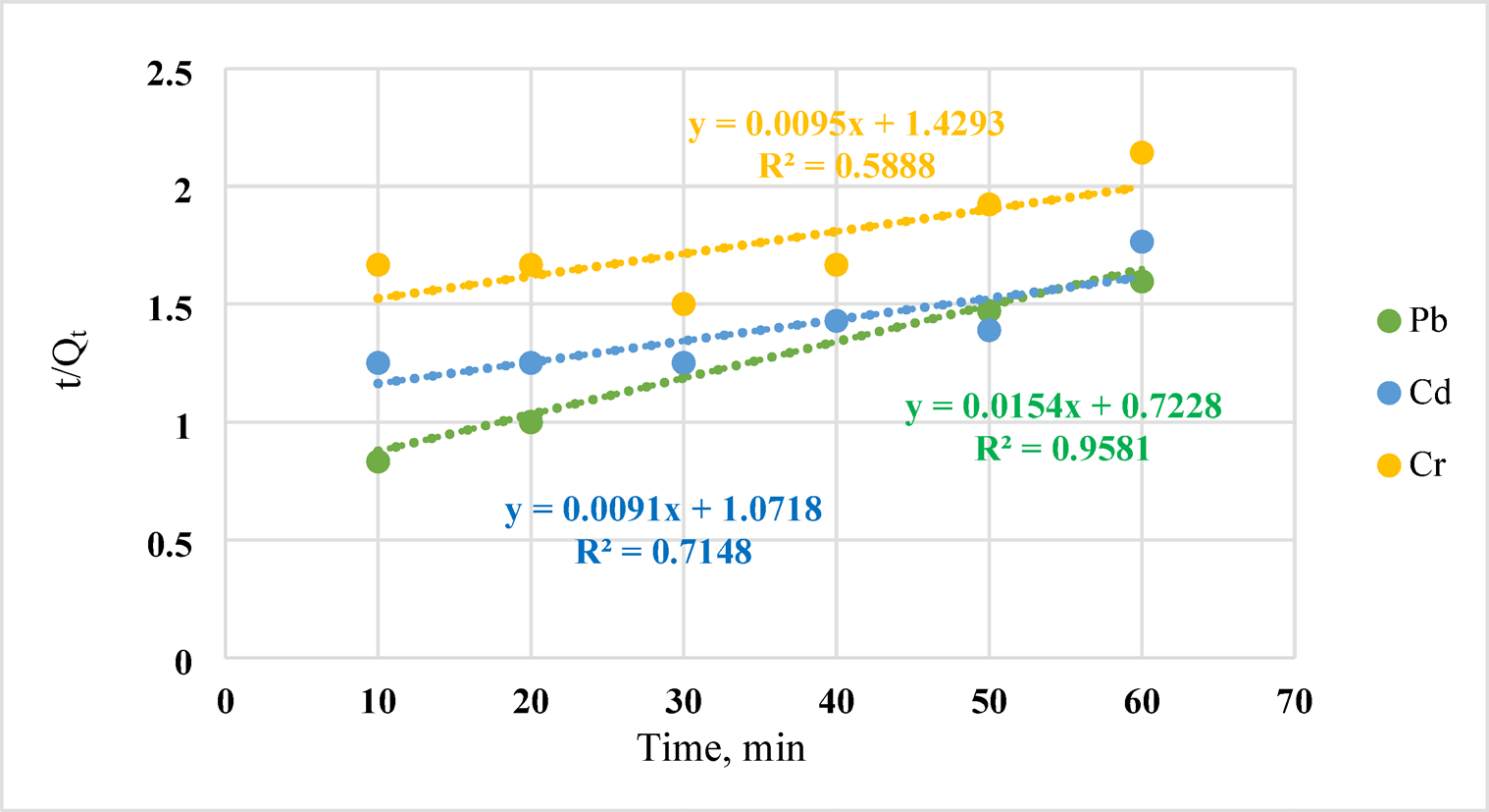
Absorption kinetic models “pseudo second order”.

The amount of metal ions adsorbed at time t (min) and the quantity adsorbed per unit mass of geopolymer at equilibrium (mg/g) are denoted by qe and qt, respectively. Two rate constants, *k*_*1*_ and *k*_*2*_, define the pseudo-first- and second-order, respectively, of a process. The kinetic characteristics of adsorption are shown in Figs. 15 and 16. The high correlation value (R^2^) indicates that the kinetics of Pb, Cd, and Cr follow pseudo-first-order behavior (Fig. 15) instead of pseudo-second-order kinetics. This proves that the features in this study that are pseudo-first-order^49^.

### Comparative study

Table 4 presents a comparative analysis of the adsorption capacities of various geopolymer from different aluminum silicate sources for Cd(II), Cr(VI), and Pb(II) ions. The results indicate that the geopolymer synthesized in this study demonstrates markedly superior adsorption capabilities for heavy metals. Specifically, the geopolymer’s enhanced performance can be attributed to its unique structural properties and the presence of reactive functional groups that facilitate stronger interactions with the metal ions. The data reveals that the geopolymer not only provides effective removal efficiencies but also emerges as a cost-effective alternative to conventional adsorbents. Its utilization of dealuminated kaolin, a by-product of aluminum sulfate industry, not only addresses waste management issues but also minimizes the overall cost of the adsorption process. These benefits make PDK-based geopolymer as an innovative solution for treating wastewater contaminated with hazardous metal ions like Cd (II), Cr (VI), and Pb (II).

**Table 4:** Comparison of adsorption capacity between different adsorbents.

Adsorbent	Adsorbate	Capacity of adsorption mg/g geopolymer	ReferenceS
Metakaolin-based geopolymer	Pb(II)	312.5	^67^
Metakaolin-based geopolymer	Pb(II)	120.5	^68^
Fly Ash	Pb(II)	166.4	^24^
Blast furnace slag-based geopolymer	Cd(II)	382.8	^69^
Coal gangue and fly ash based geopolymer	Pb(II)	85.67	^70^
Fly-ash geopolymer	Cr(VI)	17.47	^71^
Partially Dealuminated metakaolin based geopolymer	Cd(II)	150	This study
	Cr(VI)	125	
	Pb(II)	105.6	

## Conclusion

In the present study, a geopolymer was synthesized from the industrial waste of aluminum sulfate production process, and its application in removal of Pb, Cd, and Cr from synthetic wastewater. The effects of various parameters such as temperature, pH, contact time, and metal ion concentration were tested to stand over the most favorable conditions for adsorption. A total of 100% removal was achieved at (pH = 6.0, temperature = 25 °C, and initial concentration = 40 ppm). The adsorption data validated Freundlich adsorption model. The values of Freundlich constant value, R^2^ were greater than 0.99 indicating the adsorption of metal ions onto the geopolymer to be highly favorable. High adsorption capacity has been achieved for Pb (II), Cd (II), and Cr (VI) (105.6 mg/g, 150 mg/g, and 125 mg/g respectively). The adsorption process followed pseudo-1st-order kinetics yielding high correlation coefficient and the adsorbed amount at equilibrium. More than 95% of adsorption was achieved at room temperature supports the effectiveness of metal ions on the geopolymer. This work helps for the reuse of the industrial waste of alum industry through the synthesis of a geopolymeric adsorbent, which was applied successfully for removal of the Pb, Cd, and Cr ions from the polluted water. It is worth mentioning that the work based on adsorption in a certain adsorbate system in batch mode. Future work should focus on geopolymer adsorbents in fixed bed, continuous and multi-component solute systems. Furthermore, besides the proof of concept in laboratory scale synthetic wastewaters, in the near future the research should validate the results on real industrial wastewaters. Although this study successfully demonstrated the effectiveness of the synthesized geopolymer for the removal of Pb, Cd, and Cr from aqueous solutions, it did not include post-adsorption characterizations such as XRD, FTIR, or XPS to investigate the detailed removal mechanisms. Future research is recommended to conduct these analyses to distinguish between adsorption, ion exchange, and precipitation mechanisms and to gain a deeper understanding of the interaction between heavy metals and the geopolymer surface. The dealuminated kaolin waste-based geopolymer materials were investigated for effective reuse in immobilization of the adsorbed heavy metals for reduction and safe disposal of such materials paper under publishing. It was recommended to conduct further analyses to distinguish between adsorption, ion exchange, and precipitation mechanisms and to gain a deeper understanding of the interaction between heavy metals and the geopolymer surface.

## Data Availability

All data generated or analysed during this study are included in this published article.
